# Implementation of Video-Based Care in Interdisciplinary Primary Care Settings at the Veterans Health Administration: Qualitative Study

**DOI:** 10.2196/52830

**Published:** 2024-04-09

**Authors:** Claudia Der-Martirosian, Cynthia Hou, Sona Hovsepian, Maia Diarra Carter, Leonie Heyworth, Aram Dobalian, Lucinda Leung

**Affiliations:** 1 Veterans Affairs Greater Los Angeles Healthcare System Center for the Study of Healthcare Innovation, Implementation, and Policy Los Angeles, CA United States; 2 Veterans Emergency Management Evaluation Center US Department of Veterans Affairs North Hills, CA United States; 3 Office of Primary Care/Patient Care Services Veterans Health Administration Washington, DC United States; 4 Office of Connected Care/Telehealth Services Veterans Health Administration Washington, DC United States; 5 Department of Medicine University of California San Diego School of Medicine San Diego, CA United States; 6 Division of Health Services Management and Policy The Ohio State University College of Public Health Columbus, OH United States; 7 Division of General Internal Medicine and Health Services Research Department of Medicine UCLA David Geffen School of Medicine Los Agneles, CA United States

**Keywords:** interdisciplinary primary care team members, NASSS framework, nonadoption, abandonment, scale-up, spread, and sustainability, primary care, telehealth, video-based care

## Abstract

**Background:**

With the rapid shift to telehealth, there remains a knowledge gap in how video-based care is implemented in interdisciplinary primary care (PC) settings.

**Objective:**

The objective of this study was to gain an in-depth understanding of how video telehealth services were implemented in PC from the perspectives of patients and interdisciplinary PC team members at the Veterans Health Administration (VHA) 2 years after the onset of the COVID-19 pandemic.

**Methods:**

We applied a positive and negative deviance approach and selected the 6% highest (n=8) and the 6% lowest (n=8) video-using PC sites in 2022 from a total of 130 VHA medical centers nationally. A total of 12 VHA sites were included in the study, where 43 PC interdisciplinary team members (August-October 2022) and 25 patients (February-May 2023) were interviewed. The 5 domains from the diffusion of innovation theory and the nonadoption, abandonment, scale-up, spread, and sustainability (NASSS) framework guided the development of the 2 study interview guides (provider and patient). We identified themes that emerged across all interviews that were associated with the implementation of video-based care in interdisciplinary PC settings, using directed-content rapid analysis of the interview transcripts. The analysis was guided by 5 a priori NASSS domains: (1) patient condition or characteristic, (2) technology, (3) adopter system, (4) health care organization, and (5) adaptation over time.

**Results:**

The study findings include the following common themes and factors, organized by the 5 NASSS domains: (1) patient condition or characteristic—visit type or purpose (eg, follow-up visits that do not require physical examination), health condition (eg, homebound or semihomebound patients), and sociodemographic characteristic (eg, patients who have a long commute time); (2) technology—key features (eg, access to video-enabled devices), knowledge (eg, how to use videoconferencing software), and technical support for patients and providers; (3) adopter system—changes in staff roles and clinical practice (eg, coordination of video-based care), provider and patient preference or comfort to use video-based care, and caregiver’s role (eg, participation of caregivers during video visits); (4) health care organization—leadership support and access to resources, scheduling for video visits (eg, schedule or block off digital half or full days), and training and telehealth champions (eg, hands-on or on-site training for staff, patients, or caregivers); (5) adaptation over time—capacity to improve all aspects of video-based care and provide continued access to resources (eg, effective communication about updates).

**Conclusions:**

This study identified key factors associated with the implementation of video-based services in interdisciplinary PC settings at the VHA from the perspectives of PC team members and patients. The identified multifaceted factors may inform recommendations on how to sustain and improve the provision of video-based care in VHA PC settings as well as non-VHA patient-centered medical homes.

## Introduction

With the rapid expansion of telehealth services since the onset of the COVID-19 pandemic, numerous studies have focused on health care clinicians’ perspectives on telehealth service implementation in primary care (PC). These studies primarily focused on satisfaction [1-4] and barriers to and benefits of telehealth services [5-24]. Regarding barriers to PC telehealth implementation, some clinicians have experienced challenges [5-14], such as technical issues with video and patient portals, privacy or confidentiality concerns, workflow and scheduling changes, low reimbursement rates, improper telecommunication infrastructure, inability to conduct physical examinations, difficulty maintaining the therapeutic relationship with patients, skill and comfort with technology, and access to technology [5-24]. However, clinicians and patients have shared several benefits of PC telehealth implementation [21,25-34], such as reducing infection and communicable disease exposure, eliminating commute time and patients’ transportation expenses, improved medication management, better evaluation of patients’ home environment, continuity of outpatient care, flexibility in scheduling appointments, and effective screening and triaging [5-9,21,25-34].

Despite its barriers and challenges, during the COVID-19 pandemic, video-based care has garnered high levels of satisfaction among health care clinicians and patients [1-4]. However, little is known about telehealth implementation at the national level, its use among interdisciplinary PC team members, and its sustainment beyond the initial phases of the pandemic. To address these knowledge gaps, the diffusion of innovation theory and the nonadoption, abandonment, scale-up, spread, and sustainability (NASSS) framework [35], an evidence-based, theory-informed, pragmatic model, are used to help understand the factors associated with the implementation of a technology-supported health care program. The NASSS framework builds on the work of the diffusion of innovation theory [36] and addresses the success of telehealth services, referred to in this study as video-based care. Guided by 5 NASSS domains and the corresponding subdomains, this study identifies the common factors (across patients, providers, and sites) that are associated with achieving fully mainstreamed implementation of video-based care.

The main objective of this study is to understand PC team members’ and patients’ perspectives on the implementation of video-based services within the Veterans Health Administration (VHA) 2 years after the onset of the COVID-19 pandemic. VHA PC is based on a patient-centered medical home model that includes interdisciplinary team members (physicians, nurse practitioners, physician assistants, nurses, social workers, clinical pharmacists, and mental health specialists) who work together to coordinate the provision of care, including video-based care. The VHA is well-suited to examine these issues given its over 2 decades of experience in video-based care [37-40] and its rapid expansion of video-based services in PC at the onset of the COVID-19 pandemic [41].

## Methods

### Study Setting, Site Selection, and Recruitment

To have a greater understanding of the interdisciplinary PC team members’ and patients’ perspectives on the use of video-based services at the VHA, we applied a positive and negative deviance approach [42,43] and selected the 6% highest (n=8) and the 6% lowest (n=8) video-using PC sites in 2022 from a total of 130 VHA medical centers nationally. For each of the 16 selected VHA sites, we contacted the medical directors and chiefs of staff through email and shared the study materials, such as the study information sheet and study approvals. We specified in the study information sheet that participation in the study was voluntary, and if they decided to participate, they could withdraw from the study at any time or refuse to answer any question. VHA medical center directors and chiefs of staff from 12 sites (6 low and 6 high) agreed to participate in the study. The 12 study sites represent all 5 US regions (3 West, 3 Midwest, 2 Southwest, 2 Southeast, and 2 Northeast), where 9 are urban and 3 are rural.

For the provider interviews, the inclusion criteria to participate was to be a member of a VHA interdisciplinary PC team at any of the 12 study sites. After receiving approval at the 12 VHA sites, we were then referred to the PC chief of staff at each site, who assisted with the recruitment process by sharing the study information sheet with their PC team members. Interested PC team members (n=53; 3-5 per site) contacted our study team to express that they wanted to participate in a 30-minute interview. Up to 3 follow-up emails were sent to schedule the study interviews. A total of 43 PC staff members (3-4 per site) were interviewed by 2 study members remotely through Microsoft Teams during August-October 2022. All interviews were audio-recorded and transcribed.

For the patient interviews, we began the recruitment process in January 2023 by randomly selecting a total of 120 VHA PC patients (10 per site) using the VHA electronic health records. The study inclusion criteria included (1) at least 1 PC visit during the past 2 years (2021-2022), (2) a valid US postal address, and (3) a phone number. We first mailed the study recruitment letters to all potential study participants, explaining the purpose of the study, that study participation was voluntary, and that all collected interview data would be kept confidential. We also explained that they had the option to opt out by calling a designated study phone number, and if they chose not to opt out, we may contact them (by phone) in 2-3 weeks to schedule a 30-minute phone interview. To minimize the burden on the 2 study members, we mailed the recruitment letters in 2 batches (60 letters in January 2023 and 60 letters in February 2023). We contacted 83 recruited patients by phone (up to 3 phone calls) with the following results: (1) 42 (51%) voice messages (no answer), (2) 7 (8%) wrong phone numbers, (3) 9 (11%) refusals, and (4) 25 (30%) completed phone interviews (2-3 per site) during February-May 2023. The response rate for patient interviews was 33% ([25/(83-7)] × 100). We stopped recruiting patients after reaching data saturation [44]. All interviews were audio-recorded and transcribed.

### Semistructured Interview Guide

For this study, 2 semistructured interview guides (provider and patient) with open-ended questions were used. Both interview guides were developed based on 5 of the NASSS domains [35] pertaining to telehealth implementation: (1) patient conditions, illness, and characteristic; (2) technology; (3) adopter system; (4) health care organization; and (5) adaptation over time (see Table 1 for details).

**Table 1:** Patient and provider interview questions linked to nonadoption, abandonment, scale-up, spread, and sustainability (NASSS) domains and subdomains.

NASSS domains and corresponding subdomains			Patient or provider		Open-ended interview questions		
**Patient condition, illness, and characteristic**							
	1A. Visit type or purpose	Patient		Why and how was VA Video Connect (VVC), (the VA video technology/software), useful for your health conditions?			
	1B. Health condition, illness, or patient type	Provider		For what types of services/visits, patients, and health conditions is VVC appropriate? Do any of the barriers or challenges to VVC use differ by patient health conditions (eg, chronic care management)?			
	1C. Sociodemographic characteristics	Patient		What is your experience with VVC? Probe: What are/were some of the challenges/barriers to using VVC?			
	1C. Sociodemographic characteristics	Provider		What are some of the challenges to using VVC? Do any of the barriers or challenges to VVC use differ by patient socio-demographics (eg, older patients, patients experiencing homelessness)?			
**Technology**							
	2A. Key features of the technology	Patient		What type of device do you use (or used; eg, iPad, iPhone, Smartphone, laptop) for a video visit? How do you connect to the internet (eg, WiFi, broadband)?			
	2A. Key features of the technology	Provider		Do you use (or have you used) any other video app other than VVC (eg, Doximity, FaceTime) to connect with patients at their homes using video? Probe: Can you elaborate your experience with other platforms compared to VVC? What new/ongoing features of technology for VVC is your clinic or medical center using to meet the ongoing needs of patients and staff?			
	2B. Knowledge about technology	Patient		Describe the steps (scheduling a VVC appointment, during the appointment, after the appointment) used to connect to VVC?			
	2B. Knowledge about technology	Provider		What type of support/and or guidance have you/your PC team received for VVC?			
	2C. Technical support	Patient		Did you receive any consults, educational trainings, or assistance from the VA or family members/caregivers for your VVC appointment (for any of the appointment phases)? Any barriers?			
	2C. Technical support	Provider		What types of technical support, guidance, or trainings have you, and/or your colleagues, in your clinic received for VVC? Probes: What recommendations do you have for additional staff support, guidance?			
**Adopter system**							
	3A. Changes in staff roles and clinical practice	Provider		What were some of the changes in provider roles/workflows and practices, care management, care coordination, team interactions with VVC? How did these changes impact clinical practice?			
	3B. Provider and patient preference or comfort	Patient or provider		How do you feel about VVC (vs phone, vs in-person)? Probe: Do you prefer using VVC? Why or why not? Did anyone help with your VVC scheduling, VVC visit, after VVC visits?			
	3C. Caregiver’s role	Provider		What are the advantages and disadvantages of using VVC from your patients’ perspectives? Did you or your team provide any consultation/help with setting up VVC visits?			
**Health care organizations**							
	4A. Leadership support and resources	Provider or leadership		What changes had to take place in your practice/clinic to implement VVC?			
	4B. Scheduling, reminders, or day of video appointment	Provider or leadership		Were there new policies and procedures you incorporated in expanding and implementing VVC? Probe: Creating new phone/video scheduling grids?			
	4C. Trainings and telehealth champions	Provider or leadership		How did you modify and/or adapt existing administrative processes and practices? Probe: Were new ones developed? What organizational changes related to VVC did you and your facility implement (eg, policies, procedures, trainings scheduling)?			
**Adaptation over time**							
	5A. Capacity to improve	Patient		Will you use VVC in the future? Will you recommend to other patients to use VVC?			
	5B. Continued access to resources	Provider		Are you planning to continue using VVC? If not, what changes should be implemented for you to consider using VVC?			
	5B. Continued access to resources	Provider or leadership		Will your clinic continue using VVC after COVID-19? How do you see video telehealth evolving for your facility?			
	5B. Continued access to resources	Patient, provider, or leadership		If you had the opportunity to change one thing during your VVC visits at the VA, what would that be? What can the VA do to improve VVC visits with primary healthcare team?			

### Study Population

A total of 43 PC team members and 25 PC patients were interviewed remotely. PC team members represented different roles in the PC team, including 16 primary care providers (PCPs), which included physicians, physician assistants, and nurse practitioners; 12 nurses; 3 clinical pharmacists; 3 social workers; 2 mental health specialists (eg, psychiatrists and clinical psychologists); 4 scheduling clerks or supervisors; and 3 health care leadership team members. A diverse group of patients with respect to age, gender, race, ethnicity, and place of residence (rural or urban) were interviewed.

### Analysis

We identified emerged themes and factors across all provider and patient interviews that were associated with the implementation of video-based care in interdisciplinary PC settings, using directed-content rapid analysis [45,46] of the interview transcripts. This analysis was based on substantive significance [47], and it was guided by 5 a priori NASSS domains (mentioned above). A structured template was created to summarize data from each interview, with 3 team members (CH, SH, and CDM) revising the template after independently analyzing a single transcript and reviewing the others’ templates for consistency. A summary matrix of the summaries was then used to consolidate all the interview findings. Key points were transposed and sorted into NASSS framework–identified themes, then reviewed and validated by 2 project team members (CH, SH, or CDM).

### Ethical Considerations

This study was part of an ongoing quality improvement effort, and hence it was exempt from review by the institutional review board at the VHA Greater Los Angeles Healthcare System. Following study protocols, audio-recorded verbal consent was sufficient for participation in the study. To protect the privacy of study participants, all collected study information was deidentified and kept confidential. Each patient interviewee received a US $30 VHA canteen voucher after completing the interview. Provider interviewees did not receive any monetary incentives since their participation in the study was during the workday.

## Results

### Overview

In this section, the emerged themes and factors from the directed-content rapid analysis of provider and patient qualitative interviews are discussed in detail. The emerged or identified themes (or identified factors) were common across all patient and provider types. Table 2 displays the study findings for each of the 5 NASSS domains and the corresponding subdomains.

**Table 2:** Factors guided by the nonadoption, abandonment, scale-up, spread, and sustainability (NASSS) framework that impact the use of video-based services in primary care settings at the Veterans Health Administration.

NASSS domain and subdomain			Specific scenarios or examples of the appropriateness of video-based primary care
**Patient condition, illness, and characteristic**			
	Visit type or purpose	Follow-up visits to address an issue that has already been examined during an in-person visit (eg, discussing side effects from a newly prescribed medication) Follow-up visits for chronic disease management (eg, diabetes and hypertension) Getting a referral to see a specialist Continuity of care for patients who relocate to another state Medication reconciliation Clinical pharmacy (eg, review new prescriptions or respond to questions) Conducting social work assessments (eg, assess patient’s home environment) Video visits are appropriate for mental health counseling Video-based care is a good fit for patients who do not need a physical exam	
	Health condition, illness, or patient type	Established patients (eg, who have been with the same clinician for many years) Patients with mobility issues (eg, homebound or semihomebound) Patients who cannot come into the clinic for various reasons (eg, substance abuse, posttraumatic stress disorder, or anxiety) Recently discharged patients from a hospital stay or urgent or emergency care Patients in palliative care COVID-19–positive patients	
	Sociodemographic characteristics	Patients who live far away or in rural areas Patients who are experiencing homelessness Patients who are working, going to school, or have family obligations Patients who would like to save money on gas and transportation cost Patients who have working or employed caregivers who need to be present during a visit Patients who have access to a private or quiet space with no disruptions	
**Technology**			
	Key features of videoconferencing technology	Video-enabled devices for patients and primary care team members (at the office or teleworking) Access to high-speed broadband and stable internet connectivity Robust network coverage at the office User-friendly, simple-to-use videoconferencing software	
	Knowledge about technology	Be able to use a video-enabled device and navigate the videoconferencing software Test the technology before using it for the first time (eg, download the app ahead of time or test the video links)	
	Technical support	IT support for primary care team members at the office or when teleworking IT support for patients Before the first video visit, the IT help desk conducts a test call with patients to ensure everything is set for a videoconferencing visit	
**Adopter system (staff, patient, and caregiver)**			
	Changes in staff roles and clinical practice	Involve primary care team members in the video-based care coordination process (eg, if a nurse visit happens, then the primary care provider joins the video-based visit) A video-based care flow that mimics the in-person care process Telehealth protocols for different departments, services, and types of care Staffing flexibility (eg, remote provider who floats or provider coverage for other clinics)	
	Clinician and patient preferences and comfort	Provides flexibility to patients and providers to connect Patient and provider preference or comfort level to use video Patients who are not distracted (eg, they are at home and not in a store or driving) Telehealth is less stressful for some patients	
	Caregiver’s role	The caregiver is present to help with the video visit (eg, troubleshoot technical issues) The caregiver can be part of the video visit if there is a need to participate (eg, provide information) The caregiver can provide emotional support during the video visit	
**Health care organization**			
	Leadership support and resources	Leadership support at all levels (eg, primary care chief of staff, clinic, or medical director) and at all service lines (eg, primary care providers, nursing, scheduling, or pharmacy) Veterans Health Administration–issued tablets or iPads sent to qualified patients Access to a private or quiet office space for providers to conduct video visits	
	Scheduling, reminders, or day of video-based appointment	Scheduling video visits involves multiple steps and need to ensure the patient is ready to connect remotely on the day of the visit Schedule or block off remote half or full days (ie, create telehealth clinic days) More availability of telehealth appointments compared to in-person appointments Timeliness of video visits compared to in-person appointments Treat a video-based visit same as an in-person visit (eg, patients should dress appropriately, prepare a list of questions, and have their medication list ready to review) Automatically enroll new patients in video-based care	
	Trainings and telehealth champions	Online trainings and resources are available for patients and providers Prioritize video-based care trainings for all, including newly hired staff and newly enrolled patients Provide hands-on and site- or team-specific training or education for all providers, staff, and patients Identify on-site staff and patient telehealth champions to assist with hands-on training or education	
**Adaptation over time**			
	Capacity to improve	Improve technical resources, personnel support, hands-on or site-specific trainings, or education about video-based care coordination and scheduling Access video links from patient’s portal that is embedded in the electronic health record system	
	Continued access to resources	Effective and widespread communication about changes in resources and new updates to the videoconferencing software or video-based care management system and options Annual video-based care training day for providers, staff, and patients to learn about updates	

### Patient Condition, Illness, and Characteristic

#### Visit Type or Purpose

Video-based care was considered most suitable for follow-ups to routine care, particularly for patients with chronic conditions. Seeing new patients and care requiring a physical assessment was preferably done in-person. A patient explained:

If I need a quick medicine change or something along those lines where I don’t need to be physically examined then I would, yes, use a video call.

Patient 18

Moreover, video visits allow providers to assess patients’ home environment.

Video appointments give much more insights to the patients’ home status, their pride, their pets... their current living conditions that we can discuss about.

Leadership 1

I do believe the best place to take care of the patient is in their home… so, the more video we could do, I think the better we would be at taking care of Veterans. We could touch them more often.

Physician 15

Video visits also allowed better medication reconciliation, as patients could just show their bottles through the video camera.

They’re at home with their pills. They can pull them all out. They don’t have to drag them into the appointment.

Clinical pharmacist 3

Video-based care is conducive for explaining new medications or laboratory results to patients. Blood pressure readings were cited as being video-appropriate, as patients usually have their own equipment and can be overseen by their clinician while taking their own blood pressure at home. Mental health specialists felt comfortable using the technology for telemental health sessions, stating the proven effectiveness and comparability of video to in-person care.

#### Health Condition, Illness, or Patient Type

For older patients with difficulty ambulating and those who depend on family members for transportation to the clinic, video-based care is a viable option. Similarly, for established patients who have been with the same PCP for many years, and for patients who cannot come to the clinic for a variety of reasons (eg, substance abuse and anxiety), video-based care might be a good alternative. Moreover, video-based care is appropriate for patients in palliative care, COVID-19–positive patients, and recently discharged patients. One patient explained:

Following up from emergency room visits or hospital stays, those are pretty easy to do over the phone or video because you should be okay. You shouldn’t have any other issues, or if you are having issues, as long as they’re not extreme and you don’t need to go back to the ER.

Patient 17

#### Sociodemographic Characteristics

Even though younger patients might be more amenable to video-based care, age is arbitrary when understanding how to use telehealth technology.

We still live in a reality where people think that elderly people are not technologically savvy, and I don’t think that is correct.

Physician 7

It’s our older population that are not working that I think we see more of them in the virtual appointments than anything.

Nurse 8

Therefore, age should not be an exclusion criterion, but it often is. A social worker further explained that, at times, it is unrealistic for patients to be expected to come into the clinic when there is no need for them to make that extra effort.

Patients with long commutes to their local VHA clinics appreciated video visits. This allowed them to save time and money on gas and transportation. For patients who are traveling within the continental United States and who need continuity of care, video-based care can be a good option. Similarly, patients who work, have family obligations or go to school and accordingly have a hard time scheduling a clinic or in-person visit found video care to be beneficial. One patient explained:

Sometimes, I’ll set my appointment when I’m picking up my son and I’m waiting outside… and it’s usually a really good time. I’m in my car and it’s very quiet.

Patient 21

### Technology

#### Key Features of Videoconferencing Technology

Access to video-enabled devices (either a smartphone or laptop), as well as high-speed broadband and stable internet connectivity, is necessary for video-based care. At times, patients living in rural areas lacked the wireless bandwidth to take video calls. Homeless-experienced or low-income patients lacked suitable equipment (eg, a smartphone) for video services, but this issue was ameliorated by the Digital Divide Consult program that provided VHA-issued iPads or tablets for qualified patients. Yet some patients who received iPads still faced barriers to video-based care and did not use them because of internet connectivity issues or a lack of broadband access.

I saw that also happen with one of my Veterans. He wouldn’t show up on his video appointments. And I know he had a VA-issued iPad.

Physician 10

#### Knowledge About Technology

Even though video-based care might be more suitable for tech-savvy patients and providers who can use video-enabled devices, it is still important for both patients and providers to conduct at least 1 test call before using the VHA video-based technology for the first time (eg, download the app ahead of time and test the video links).

#### Technical Support

IT support is available for PC team members at the office or when teleworking. IT support for patients is also available, which can help patients ensure everything is prepared for their upcoming videoconferencing visit. A video test call can be done with the IT help desk or a live web-based chatbot to ensure patients’ devices are compatible with the videoconferencing software, but often these services are underused by patients.

### Adopter System (Staff, Patient, and Caregiver)

#### Changes in Staff Roles and Clinical Practice

There have been challenges in video-based care coordination, as some aspects and processes of in-person visits are not easily transferable to video-based visits. For example, after a nurse conducted the initial video-based check-in (eg, going over vitals and the patient’s medication list), the video call was dropped during the transfer, and the PCP was unable to join the video visit. Creating processes and procedures to manage video-based care coordination among PC team members is key for providing team-based video care.

Additionally, video-based care can provide staffing flexibility. For example, video-based providers float and provide coverage at other clinics. Regarding clinical practice, there is a need to set up video-based care protocols and guidelines for different departments, services, and types of care, so there is guidance as to when video visits are appropriate.

#### Provider and Patient Preference or Comfort

Video-based visits provide flexibility for patients and providers to connect remotely. Provider and patient preferences regarding video-based care are important factors, given that there were strong opinions among providers against video-based care.

We have some providers that refuse to conduct video appointments… they just don’t like change.

Nurse 5

Regarding patient preference and comfort coming into the clinic, video-based care offers a less stressful option for patients to connect with their providers. One patient explained:

I was back in the comfort of my personal space where I feel safe, and I can open up and talk better than at an office where you’re not as comfortable because it’s not your space… And I feel that I was able to open up more and really benefit from the mental healthcare than when I go to the office because I’m already like stressed because I’m there and I’m in a hospital environment and I don’t like hospitals at all.

Patient 23

However, there was a concern with distracted patients during video visits.

Sometimes the patient does not engage enough with you, or they get distracted by the environment.

Physician 2

They might log on, but they’re busy doing their hair. It’s almost like they’re face timing of a friend or a family member.

Mental health specialist 03

Overall, patients preferred occasional in-person visits to feel like they were receiving adequate attention and care. Generally, patients have strong opinions either for or against video-based care.

The ones that wanted to do it, it was a really good process. The ones that didn’t want to do it didn’t want to hear anything about it.

Nurse 5

#### Caregiver’s Role

In some instances, it is important for caregivers to be present during the visit and be involved in the patient’s care, such as providing emotional support, having a better understanding of the health condition, and sharing information about what the patient is going through. Video-based visits can provide more opportunities for caregivers to get more involved with patient care. One patient explained:

I think it’s wonderful because it gave my wife a better feel for what was going on with me.

Patient 14

Another patient shared:

I did have one session with my husband because I was trying to get him qualified as a caregiver. So, we had a video appointment for that. It was very convenient and a great option because, at the time, I was bedridden with a back injury before I had back surgery. And he was pretty much taking full time care of me to get me out of bed, showered, dressed, the whole thing. It was very helpful to have that option that we could all meet and have that evaluation done.

Patient 22

In another instance, a social worker explained:

They’d [adult son] rather just schedule that time to be at their parent’s house and sign into the video than come in person.

Social worker 3

### Health Care Organization

#### Leadership Support and Resources

Leadership support at all levels (eg, PC chief of staff and clinic or medical director) and from all departments (eg, PCPs, nursing, scheduling, and pharmacy) is key for the successful implementation of video-based care in PC. Additionally, having a designated physical space (eg, a private quiet space with no distractions or interruptions) and computer hardware, such as video-enabled devices, are a necessity for the successful implementation of video-based care.

#### Scheduling, Reminders, and Day of Video Appointment

Before scheduling a video-based PC visit, schedulers or clerks ask patients a series of questions about the types of devices they own to determine if a video visit is feasible. The scheduler or clerk also sends the patient the video link and creates the follow-up appointment. Before the day of the video appointment, reminders through SMS text messages as well as email are sent. Automatically enrolling new patients for video visits is also an option, which makes scheduling for video-based visits easier. In-person or clinic visits are still the most frequently scheduled across clinics. However, after the provider puts in the video request that the patient agreed to, some schedulers or clerks failed to follow through with scheduling the video-based appointment.

Video clinics are being underutilized, not because of lack of patient interest. It’s being underutilized because schedulers are not calling patients to make the appointment in the video clinic.

Physician 2

There is also the option to block off half or full days for video visits. Patients shared that video visits were more timely and easier to schedule compared to in-person appointments. Video-based visits should be treated just the same as an in-person visit. As such, patients need to dress appropriately, prepare a list of questions, have their medication list ready to review and find a quiet place for the video call.

#### Trainings and Telehealth Champions

Video-based care training for PC team members is done through formal web-based training. However, hands-on training or trial-and-error is the best way to learn how to use the VHA video technology.

I think the best way people can walk somebody else through it is if they do it themselves.

Physician 13

Training should be tailored for each clinic and team member, so each can understand their role in the video-based care process. VHA leadership noted that they combined multiple video training courses into 1 web-based course for new providers. An optional 1-day intensive training course is also available. Ultimately, having a telehealth champion who provides hands-on, personalized training and updates to PC team members can be critical to effectively educating the PC team on how to use video-based care.

We did have a super user in the clinic who was able to go in and show everyone how to do it [ie, use VA video technology].

Nurse 3

For patients, training for video-based visits usually falls on the nurses, schedulers, clerks, technicians, or other clinic staff who enroll patients in video-based scheduling for the first time. Having a patient telehealth champion, for example, a veteran sharing how they benefited from a video visit, might be helpful in providing peer-to-peer mentoring and promoting video-based visits. Overall, it seems that patients choose to have more telehealth appointments after being trained.

Once we get it set up for them [patients] and they feel comfortable with the whole [thing]—they’re utilizing it [VA video technology] quite a bit.

Nurse 8

### Adaptation Over Time (Capacity to Improve and Continued Access to Resources)

Suggestions for improving video-based care at the VHA centered around 5 main areas: technological resources, personnel support, training, care coordination, and scheduling. Regarding resources, video-based care should be connected to the patient’s electronic health care records for easier access to the web-based appointment link. Accessibility of video on cellular devices, like using SMS text messages to quickly communicate with patients. There is a need to hire more clerical staff to assist with video-based care scheduling and technical support (eg, a telehealth enrollment coordinator role on the team or a telehealth help center to assist veterans with technical issues). Beyond personnel support, there is a need for more physical space in clinics, so they could have privacy to conduct telehealth visits. Other suggestions included having more resources to support video technology, such as choosing site-specific internet networks and improving network coverage overall.

Recommendations to improve training focused on having nonclinical staff administer patient education in group and in-person settings. Patients need adequate training to use VHA video technology.

We need to teach them. We need to take the time and put the investment… but too often, we blame the patient, I think inappropriately.

Physician 11

When asked about care coordination, the video care process should mimic the flow of in-person care while striving for “care management and the same depth of treatment.”

Regarding scheduling, providers asked for separate days designated for video and face-to-face appointments. It is important to have 1 centralized platform or web-based grid where all types of appointments, telehealth and in-person, can be scheduled. Schedulers or clerks should have a script or checklist to ensure that patients understand their video-based care options. Finally, site-specific telehealth management is needed, acknowledging that regional differences and specific site needs may affect each VHA clinic’s telehealth or video adoption and implementation.

## Discussion

### Overview

In this study, the 5 NASSS domains that pertained to the implementation of telehealth services guided the qualitative analysis and identified key factors associated with the implementation of video-based services in PC from interdisciplinary team members’ and patients’ perspectives 2 years after the onset of the COVID-19 pandemic. In terms of patient condition, illness, and characteristics, the study findings concurred with previous studies that video-based care is most appropriate for follow-up visits that do not require physical examinations, and in some cases, it might be well-suited for follow-up visits for chronic care management [48,49]. This study corroborated findings from previous studies that video visits are appropriate for semi- or fully homebound patients with mobility issues who benefit from having a caregiver present at their medical appointments [50,51]. Similar to previous studies, convenient access to video-based care was needed for patients with full-time jobs, family obligations, transportation difficulties, and long travel distances for in-person care [5-24]. Regarding age, the study findings illustrated that it is important not to assume that older patients are disinterested or not qualified in using video-based care.

This study focused on video-based care; therefore, the key features of videoconferencing technology, knowledge on how to use the VHA video technology, as well as the availability of technical support, were key factors in supporting video-based care at the VHA. This study did not examine the extent to which phone was the preferred mode of video-based care as compared to video or the extent to which video visits switched to phone visits because of technical issues or other barriers. A recent study of 4691 Medicare beneficiaries [52] found that phone visits were more common when patients were given the option to use video or phone, especially among those with less access to technology and information about telehealth services. Therefore, future studies should examine the extent to which it is important to offer both options of video-based care, phone (audio-only), or video, while also addressing technological barriers to video use.

Regarding the adopter system, the study findings illustrated that there are challenges for video-based care coordination to mirror (or mimic) the workflow of routine (in-person) care (eg, the nurse conducts the initial part of the video visit and then the PCP joins the video visit). Accordingly, more research is needed to identify better ways of integrating all PC team members in the coordination and provision of video-based visits. Additionally, as illustrated in previous studies [5-24], this study also found that patient and provider preferences, as well as comfort level, influence the use of video-based care. As stated previously [25-34], video-based care offers providers and patients flexibility. During video visits, patients have the flexibility to not only connect from the comfort of their homes but to also include their caregivers during video visits if needed or desired.

For health care organization factors, leadership support at clinic and departmental levels is needed to provide the resources necessary for the successful implementation of telehealth services. Additionally, the role of schedulers or clerks in video care is critical, especially for a large integrated health care system such as the VHA. Schedulers or clerks help introduce patients to video-based care options and assist patients in navigating the VHA web-based platform. Additional research is needed to identify best practices to improve the scheduling process of video-based visits. In terms of trainings, in addition to VHA web-based video-based care trainings, which are available to all PC team members at the national level, it is still important to have patient and provider telehealth champions at the local site or clinic level who can encourage use and provide peer-to-peer, hands-on, site- or clinic-specific trainings.

Study participants shared many areas of improvement that can help sustain telehealth adaptation over time. Like previous studies [53], the study findings highlighted that it is important for health care organizations to have the capacity to improve technological resources, personnel support, training, and video-based care coordination and continue to provide access to these resources. In addition, the study findings also highlight the importance of improving scheduling processes and platforms to better meet the needs of all providers and patients. Finally, it is important to acknowledge that site-specific telehealth management is needed, since regional differences and specific site needs may affect how each VHA clinic implements telehealth or video-based care in PC settings.

### Limitations

The major strengths of this study are the following: first, the study included 12 VHA medical centers located in geographically diverse settings. Second, these study sites, including 9 urban and 3 rural, had varying rates of video use, ranging from lowest to highest. Third, the directed-content rapid analysis, guided by a priori NASSS domains, identified emerged themes or factors across all interviews (43 PC team members and 25 PC patients). Despite this, the study has several limitations. First, this study did not examine the differences between patient types, appointment types, provider types, and sites. Instead, the factors that were identified in this study were common across all patients, all PC team members, and all sites. Future studies should consider which factors differentiate between sites (eg, urban vs rural), provider types (eg, mental health specialists vs PCPs), patient types (eg, male vs female and with diabetes vs without diabetes), as well as appointment types (mental health visit vs PC visit). Second, since this study did not use probability sampling strategies to select the study sites or the provider study sample, the generalizability of the study findings to all VHA PC clinics and providers is limited. Furthermore, even though simple random sampling was used to select the patient study sample, the study’s main purpose was to have an in-depth understanding of the implementation process of video-based care at the selected VHA study sites. Hence, the generalizability of the study findings to all VHA patients is limited. Third, the generalizability of study findings may be limited in non-VHA health care systems for various reasons, including that VHA clinicians do not have the same cross-state licensure restrictions, especially since the passage of the Anywhere-to-Anywhere Act (in May 2018) [54,55], where VHA expanded telehealth services by allowing health care clinicians to treat patients across state lines; and VHA has a capitated payment system, which makes it easier to implement telehealth since it is not subject to third-party payer arrangements [54]. However, recent COVID-19 telehealth waivers have increased non-VHA health care providers’ and clinics’ telehealth capability, such as allowing telehealth services across state lines. Therefore, there are more similarities now between VHA and non-VHA telehealth services than even in the recent past. As such, study findings may still be applicable to non-VHA clinical settings and contribute to the growing evidence base surrounding factors most salient to the successful implementation of telehealth services at PC clinics.

### Conclusions

Given that VHA PC is based on a patient-centered medical home model that includes interdisciplinary team members who work together to coordinate the provision of care, including video-based care, we examined the implementation of video-based care in interdisciplinary PC settings from the perspectives of PC team members as well as patients at 12 different VHA health care settings (9 urban and 3 rural). Guided by 5 a priori NASSS domains and the corresponding subdomains, we identified common factors (across patients and PC team members) that were associated with the implementation of video-based care in interdisciplinary PC settings. The identified multifaceted factors that resulted from the qualitative analysis of the collected interview data may help inform recommendations on how to sustain and improve video-based care in VHA PC settings and other non-VHA patient-centered medical homes.

## Abbreviations

NASSS: nonadoption, abandonment, scale-up, spread, and sustainability
PC: primary care
PCP: primary care providers
VHA: Veterans Health Administration

## Data Availability

The data sets that were generated and analyzed during this study are not publicly available because the collected information might compromise the privacy of the study participants. The data that support the findings of this study are available from the corresponding author (CD-M) on reasonable request.
